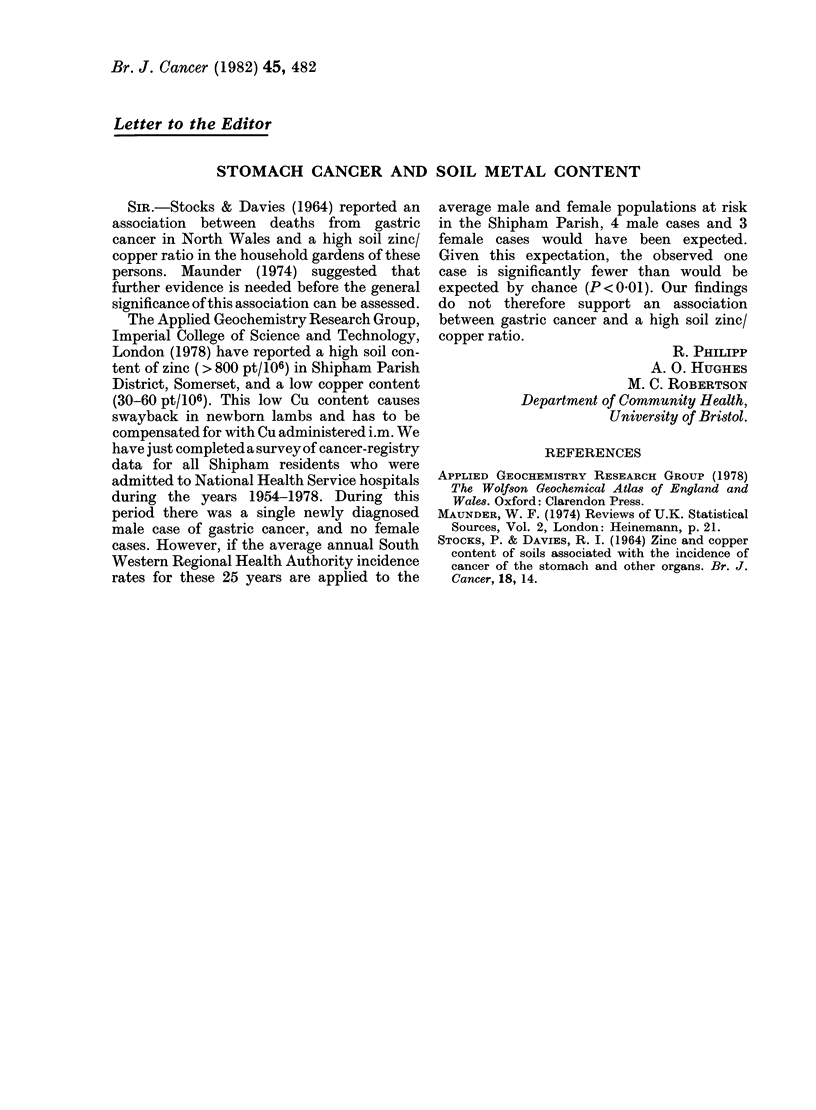# Stomach cancer and soil metal content.

**DOI:** 10.1038/bjc.1982.79

**Published:** 1982-03

**Authors:** R. Philipp, A. O. Hughes, M. C. Robertson


*Br. J. Cancer* (1982) **45**, 482

### *Letter to the Editor*

### STOMACH CANCER AND SOIL METAL CONTENT

SIR.—Stocks & Davies (1964) reported an association between deaths from gastric cancer in North Wales and a high soil zinc/copper ratio in the household gardens of these persons. Maunder (1974) suggested that further evidence is needed before the general significance of this association can be assessed.

The Applied Geochemistry Research Group, Imperial College of Science and Technology, London (1978) have reported a high soil content of zinc ($>800$ pt/$10^6$) in Shipham Parish District, Somerset, and a low copper content (30–60 pt/$10^6$). This low Cu content causes swayback in newborn lambs and has to be compensated for with Cu administered i.m. We have just completed a survey of cancer-registry data for all Shipham residents who were admitted to National Health Service hospitals during the years 1954–1978. During this period there was a single newly diagnosed male case of gastric cancer, and no female cases. However, if the average annual South Western Regional Health Authority incidence rates for these 25 years are applied to the average male and female populations at risk in the Shipham Parish, 4 male cases and 3 female cases would have been expected. Given this expectation, the observed one case is significantly fewer than would be expected by chance ($P<0{\cdot}01$). Our findings do not therefore support an association between gastric cancer and a high soil zinc/copper ratio.

R. PHILIPP
A. O. HUGHES
M. C. ROBERTSON
*Department of Community Health,*
*University of Bristol.*

REFERENCES

APPLIED GEOCHEMISTRY RESEARCH GROUP (1978) *The Wolfson Geochemical Atlas of England and Wales.* Oxford: Clarendon Press.

MAUNDER, W. F. (1974) Reviews of U.K. Statistical Sources, Vol. 2, London: Heinemann, p. 21.

STOCKS, P. & DAVIES, R. I. (1964) Zinc and copper content of soils associated with the incidence of cancer of the stomach and other organs. *Br. J. Cancer*, **18**, 14.